# Medication review and deprescribing in different healthcare settings: a position statement from an Italian scientific consortium

**DOI:** 10.1007/s40520-023-02679-2

**Published:** 2024-03-08

**Authors:** Massimo Carollo, Virginia Boccardi, Salvatore Crisafulli, Valeria Conti, Paola Gnerre, Simonetta Miozzo, Emanuela Omodeo Salè, Fabio Pieraccini, Mauro Zamboni, Alessandra Marengoni, Graziano Onder, Gianluca Trifirò, the Italian Scientific Consortium on medication review, deprescribing, Raffaella Antonioni, Margherita Selleri, Giacomo Vitturi, Amelia Filippelli, Salvatore Corrao, Gerardo Medea, Alessandro Nobili, Luca Pasina, Emanuela Omodeo Salé, Francesco Maria Petraglia, Elisabetta Poluzzi, Alessandro Valle, Adriano Vercellone, Nicola Veronese

**Affiliations:** 1 Department of Diagnostics and Public Health, University of Verona, P.Le L.A. Scuro 10, 37124 Verona, Italy; 2 Institute of Gerontology and Geriatrics, Department of Medicine and Surgery, University of Perugia, Perugia, Italy; 3 Clinical Pharmacology Unit, Department of Medicine, Surgery and Dentistry, University of Salerno, Salerno, Italy; 4 Ligure 2 Local Health Unit, Savona, Italy; 5 Italian Society of General Medicine and Primary Care, Florence, Italy; 6 Division of Pharmacy, IEO European Institute of Oncology IRCCS, Milan, Italy; 7 IRCCS Centro Cardiologico Monzino, Milan, Italy; 8 Romagna Local Health Unit, Pharmacy Unit, Forlì-Cesena, Italy; 9 Department of Medicine-Geriatric Division, University of Verona, Verona, Italy; 10 Department of Clinical and Experimental Sciences, University of Brescia, Brescia, Italy; 11 Università Cattolica del Sacro Cuore, Rome, Italy; 12 Fondazione Policlinico Gemelli IRCCS, Rome, Italy

**Keywords:** Medication review, Deprescribing, Frailty, Multimorbidity, Patient-centered care, Polypharmacy

## Abstract

Recent medical advancements have increased life expectancy, leading to a surge in patients affected by multiple chronic diseases and consequent polypharmacy, especially among older adults. This scenario increases the risk of drug interactions and adverse drug reactions, highlighting the need for medication review and deprescribing to reduce inappropriate medications and optimize therapeutic regimens, with the ultimate goal to improving patients’ health and quality of life. This position statement from the Italian Scientific Consortium on medication review and deprescribing aims to describe key elements, strategies, tools, timing, and healthcare professionals to be involved, for the implementation of medication review and deprescribing in different healthcare settings (i.e., primary care, hospital, long-term care facilities, and palliative care). Challenges and potential solutions for the implementation of medication review and deprescribing are also discussed.

## Introduction

Recent advances in medical treatments for chronic diseases have led to longer life expectancy, resulting in a considerable increase of the number of patients with multimorbidity, i.e., the coexistence of two or more chronic diseases [1]. One of the most direct consequences of multimorbidity is polypharmacy, which is most often defined as daily regular intake of five or more different medications, common practice especially in older adults [2]. Based on this definition, prescribing medicines without an evidence-based indication, as well as medicines that are no longer effective or pose a risk for adverse drug reactions (ADRs) should be considered as inappropriate polypharmacy.

According to the last Italian National Report on Medicine Use, issued by the Italian Medicines Agency (AIFA), approximately two thirds of adults aged 65 and more are on polypharmacy, with about 25% of them taking daily at least ten different medications [3]. It is well known that polypharmacy increases the risk of drug–drug interactions (DDIs), which in turn may cause ADRs, in both hospital and outpatient settings [4]. In addition, patients on polypharmacy have higher risk of receiving potentially inappropriate medications (PIMs), i.e., drugs for which either the risk of ADRs outweighs expected benefit, or there is not enough scientific evidence on benefits or there are more effective and safer therapeutic alternatives [5].

In all healthcare settings, a large body of evidence documented that reducing potentially inappropriate prescriptions (PIPs) lowers the risk of ADRs and related emergency department visits, hospitalizations, prolonged hospital stays, and increased healthcare costs. Therefore, it is crucial to implement preventive strategies to reduce PIPs and to simplify patients’ daily therapeutic regimens. Medication review and deprescribing is a two-step patient-centred approach aiming at optimising the use of medicines through a systematic and periodic evaluation of pharmacological therapies (medication review) received by a patient and the judicious withdrawal or dose reduction of medications that are either inappropriate or unnecessary (deprescribing) [6]. Medication review can be categorized into different levels: unstructured and/or opportunistic (ad hoc, level 0), technical review of list of patient’s medicines (prescription review, level 1), review of medicines with patient’s full notes (treatment review, level 2), face-to-face review of medicines and condition (clinical medication review, level 3) [7].

The primary objectives of medication review and deprescribing are to enhance the benefit-risk profile of pharmacological treatments, improve adherence to appropriate chronic therapies, and ultimately promote the patient's overall health and quality of life [8]. To optimize polypharmacy, it is necessary to assess the patient’s medication list, identifying both unnecessary drug prescriptions (overtreatment) as well as potential undertreatment. This evaluation should consider individual patients’ care goals, clinical conditions, life expectancy, and preferences [6].

In 2016, the National Institute for Health and Clinical Excellence (NICE) issued the guidelines for the clinical and healthcare management of adults with multimorbidity and polypharmacy [9]. These guidelines were subsequently adapted to the Italian context by a panel of experts from several scientific societies, who updated specific clinical questions and identified new questions of national interest. In 2022, the “Inter-Society Guideline for the Management of Multimorbidity and Polypharmacy” was published in the National Guidelines System of the Italian National Institute of Health, outlining, among other topics, the general principles for deprescribing specific drug classes (i.e., anti-hypertensive drugs, proton pump inhibitors [PPIs], statins, and antiplatelet agents) [1]. However, applying these recommendations in clinical practice remains a challenge. Specifically, the challenges of the medication review and deprescribing mainly concern the structure of the service, the methodologies and tools to be used during the intervention phases, and the evaluation of its clinical and economic impacts. In response to these challenges, a multidisciplinary team of experts from the main Italian national scientific societies in the fields of pharmacology, geriatrics, internal medicine, and general medicine was set up to define evidence-based operational strategies for the implementation of medication review and deprescribing in various healthcare settings. In detail, experts from the following scientific societies were involved:

- The Academy of Geriatrics (*Accademia di Geriatria*, AG)
- The Federation of Associations of Hospital Doctors on Internal Medicine (*Federazione delle Associazioni dei Dirigenti Ospedalieri Internisti Medicina Interna*, FADOI)
- The Italian Society of Palliative Care (*Società Italiana di Cure Palliative*, SICP)
- The Italian Society of Clinical Pharmacy and Therapeutics (*Società Italiana di Farmacia Clinica e Terapia*, SIFACT)
- The Italian Society of Hospital Pharmacy (*Società Italiana di Farmacia Ospedaliera*, SIFO)
- The Italian Society of Pharmacology (*Società Italiana di Farmacologia*, SIF)
- The Italian Society of Hospital and Community Geriatrics (*Società Italiana di Geriatria Ospedale e Territorio*, SIGOT)
- The Italian Society of Gerontology and Geriatrics (*Società Italiana di Gerontologia e Geriatria*, SIGG)
- The Italian Society of General Medicine and Primary Care (*Società Italiana di Medicina Generale e Delle Cure Primarie*, SIMG)
- The Italian Society of Internal Medicine (*Società Italiana di Medicina Interna*, SIMI).

## Aim

To describe key elements, tools, timing, healthcare professionals to be involved, and main issues concerning polypharmacy to be evaluated for the implementation of medication review and deprescribing in different healthcare settings.

## Target population

Medication review and deprescribing are intended primarily, but not exclusively, for older adults (i.e., aged ≥ 65 years) on polypharmacy and, more generally, for all patients deemed eligible by the physicians, based on the complexity of the pharmacological therapies and the vulnerability of the patients themselves. In this context, frail patients, despite chronological age, represent a vulnerable population within the healthcare landscape. Due to higher susceptibility to adverse health outcomes, these adults often struggle with diminished physical resilience, functional limitations, and multiple comorbidities. Addressing the unique needs of frail patients is a crucial aspect of modern healthcare, aiming to optimize their health and ensure quality of life [6].

The “Inter-society Guideline for the Management of Multimorbidity and Polypharmacy” identified several frailty algorithms that can be useful in identifying the patients most at risk of ADRs and adverse events such as falls, and thus preferential targets of medication review and deprescribing [1].

## Methodology of medication review and deprescribing

The patient's evaluation should be multidimensional, followed by a multidisciplinary approach, and the management of patients' clinical data must be conducted in compliance with data protection regulations for sensitive information [8].

The healthcare professionals who should be involved in the service include physicians in charge of the patient’s care, e.g., general practitioners (GPs), ward physicians, and specialists, then clinical pharmacologists (both pharmacists and physicians), hospital pharmacists with training and experience in clinical pharmacology, and other supporting healthcare professionals, such as nurses and psychologists [10].

**Fig. 1:**
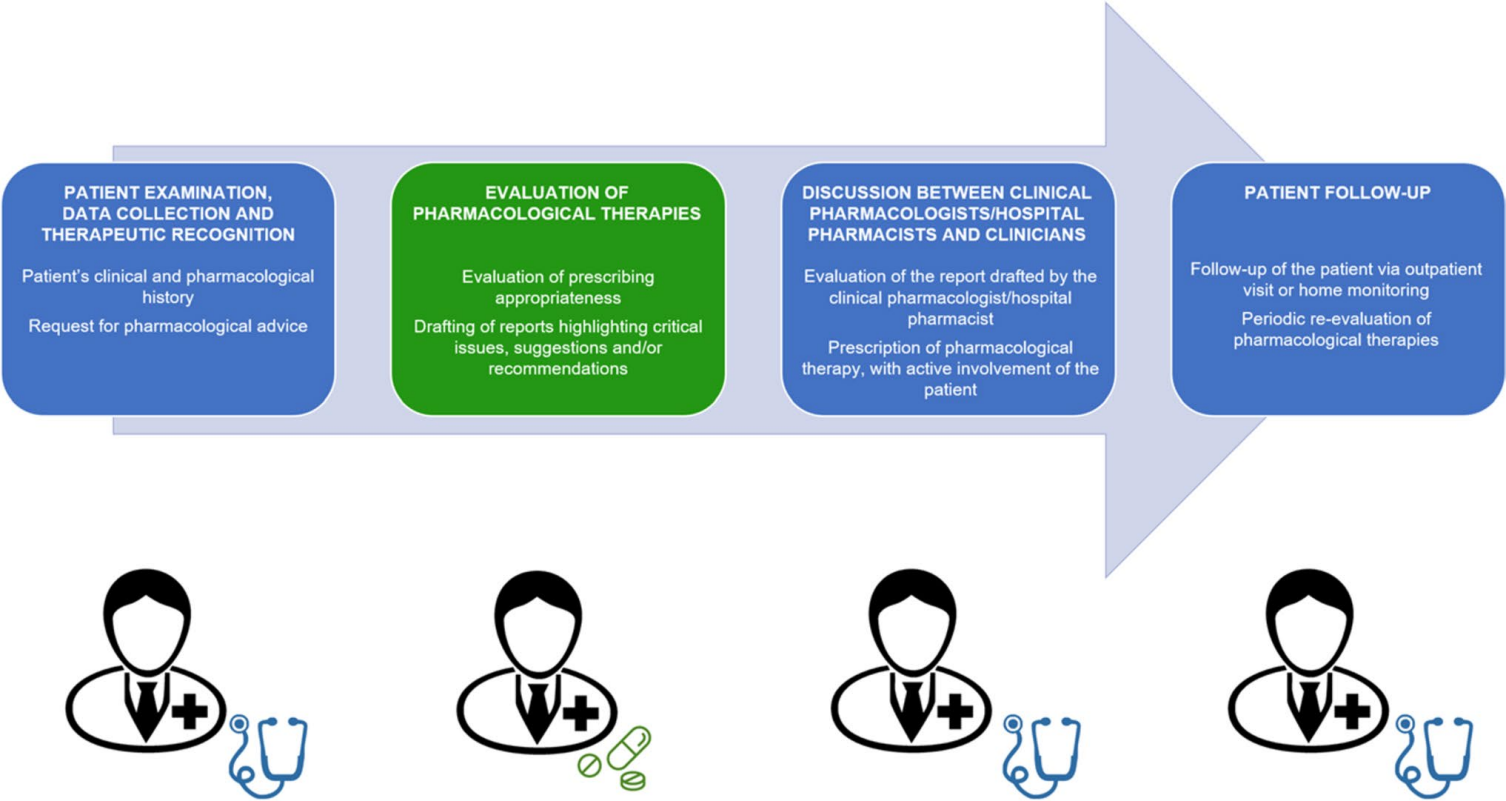
Main activities of a Medication Review and Deprescribing Service

In general, the process should rely on validated tools and algorithms, and be structured in four sequential phases (Fig. 1): 1. pharmacotherapeutic history; 2. pharmacotherapeutic analysis; 3. multidisciplinary pharmacotherapeutic plan; and 4. follow-up [1, 6, 8, 10].

### Pharmacotherapeutic history

The initial phase of medication review and deprescribing involves the clinical evaluation of the patient, which includes a comprehensive medical and pharmacological history. Regarding the medical history, the main elements to assess, which may partly vary across different healthcare settings, include the evaluation of anthropometric parameters (e.g., weight, height, and body mass index), liver and kidney function, comorbidities that may increase substantially the complexity of the therapeutic regimens and require special monitoring (e.g., heart failure, chronic kidney disease, diabetes mellitus, chronic respiratory diseases, neurodegenerative diseases, and malignancies), results of laboratory (e.g., hemoglobinemia, electrolytes serum level) or diagnostic-instrumental tests (e.g., QTc prolongation), relevant for the assessment of patient clinical conditions and/or the effectiveness and safety of prescribed therapies, and multidimensional/functional assessment scales, to be selected based on the healthcare setting. In addition, social and environmental factors (e.g., presence of caregiver) may be taken into account.

Regarding the pharmacological history, a complete list of the medications taken by the patient should be retrieved, including over-the-counter medications and dietary supplements. In case of poor patient cooperation, a relative/caregiver may be consulted. The elements to be retrieved and carefully evaluated for each drug taken should encompass indications of use, daily dosage, frequency of administration, duration and start date of therapy, level of adherence to chronic therapies, complexity of therapeutic regimens, evaluated using dedicated tools such as the Medication Regimen Complexity Index, and other factors including documented lack of effectiveness, suspected ADR attributable to one or more of the concomitant medications, and history of drug allergies or ADRs.

### Pharmacotherapeutic analysis

The second phase of medication review and deprescribing is the assessment of specific items of polypharmacy, using validated tools/source of information: inappropriate daily dosage and duration, clinically relevant DDIs, risk of ADRs, prescribing cascade, presence of PIPs. As regards PIPs, particular attention should be paid to the use of drugs contraindicated for age or presence of comorbidities, drugs prescribed in absence of an indication of use (e.g., use of PPIs as gastroprotection in patients who discontinued antiplatelet treatment) or drugs for which the expected benefit is null or marginal (e.g., use of statins in primary prevention in patients with limited life expectancy). Moreover, another factor to evaluate is the overall anticholinergic burden of prescribed drugs, especially in older and frail patients with already documented cognitive decline. Table 1 lists several algorithms, criteria, tools, and information sources that can be used to perform these assessments. The pharmacological classes (and related indication of use and potential side effects to be monitored) that most often require careful re-evaluation in elderly patients are described in Table 2.

**Table 1:** Tools and information sources available for medication review and deprescribing

	Tools and information sources
Predictive algorithms	Medication Appropriateness Index (MAI)
	The Assess, Review, Minimize, Optimize, Reassess tool (ARMOR)
	The Good Palliative-Geriatric Practice algorithm
	Prescribing Optimization Method
	NHS Scotland 7 Steps to Appropriate Polypharmacy: Aim, Need, Need, Effectiveness, Safety, Efficiency, Patient-centred
	Need and indication, Open questions, Tests and monitoring, Evidence and guidelines, Adverse events, Risk reduction or prevention, Simplification and switches (NO TEARS)
	5-Step deprescribing framework
	The Deprescribing Rainbow
	Decision Making for Older Adults With Multiple Chronic Conditions
Drug-specific algorithms	Proton Pump Inhibitor Deprescribing Algorithm
	Antihyperglycemics Deprescribing Algorithm
	Antipsychotic (AP) Deprescribing Algorithm
	Benzodiazepine & Z-Drugs (BZRAs) Deprescribing Algorithm
	Cholinesterase Inhibitor (ChEI) and Memantine Deprescribing Algorithm
	NSW Therapeutic Advisory Committee (TAG) Deprescribing guides
	Web Portal Software Anticholinergic Burden Calculator
Criteria or lists of potentially inappropriate medications	Beers Criteria
	Screening Tool to Alert Doctors to Right Treatments (START)
	Screening Tool of Older Persons’ potentially inappropriate Prescriptions (STOPP)
	Screening Tool of Older Persons Prescriptions in Frail adults with limited life expectancy (STOPPFrail)
	Fit fOR The Aged (FORTA) List
	Improved Prescribing in the Elderly Tool (IPET)
	PRISCUS list
	List of Evidence-baSed depreScribing for CHRONic patients (LESS-CHRON)
	The Norwegian General Practice—Nursing Home criteria (NORGEP-NH)
	European Union (EU) (7)—Potentially Inappropriate Medications (PIM) list
Questionnaires	Revised Patients’ Attitudes Towards Deprescribing (rPATD) Questionnaire
	Patient Perceptions of Deprescribing (PPoD) survey
Guidelines	Guidelines for the Management of Multimorbidity and Polypharmacy
	A practical guide to stopping medicines in older people (Best Practice Journal 2010; 27:10–23)
	Palliative and Therapeutic Harmonization Program (PATH)
Medication review web applications	MedStopper (https://medstopper.com/)
	INTERCheck (https://intercheckweb.marionegri.it/)
	Drug-PIN (https://www.drug-pin.com/)
	Medicines Complete (https://www.medicinescomplete.com/log-in/)
Interaction checkers	INTERCheck
	Lexicomp
	Micromedex
	Drugs.com
	Drug-PIN
	MedicinesComplete

**Table 2:** Drug classes that most frequently require re-evaluation in elderly patients, with related indications and main adverse drug reactions to monitor

Pharmacological class	Indication	Adverse drug reactions/events
Benzodiazepines	Insomnia, anxiety	Daytime drowsiness, falls, confusion, dependence, paradoxical effect (increased anxiety, insomnia, irritability, worsening of seizures for epileptics), car accidents, cognitive decline
Antiarrhythmics	Cardiac arrhythmias	Bradycardia, arrhythmias, electrolyte imbalances
Anticoagulants	Thrombotic or ischemic conditions	Haemorrhages, thrombosis
Antipsychotics	Psychiatric and behavioural symptoms	Weight gain, sedation, memory loss, and involuntary movements
Antidepressants	Depression, anxiety	Drowsiness, gastrointestinal disorders, and weight gain, prolonged QTc, electrolyte disorders
Antihypertensives, including diuretics	High blood pressure	Electrolyte imbalances, dehydration, acute renal failure, hypotension, dizziness, falls, pre-syncope, syncope
Antidiabetics	Diabetes	Hypoglycemia, weight gain/loss, gastrointestinal disorders, peripheral edema, urinary tract infections
Bisphosphonates, vitamin D, and calcium	Fracture prevention	Gastrointestinal disorders, muscle pain, headache, and atypical fractures
Non-steroidal anti-inflammatory drugs	Inflammatory conditions, pain	Gastrointestinal ulcers, gastrointestinal bleeding, and acute renal failure
Proton pump inhibitors	Gastric ulcer, gastroesophageal reflux	Diarrhea, headache, increased risk of gastrointestinal infections, renal failure, bone fractures, vitamin B12 and other nutrient deficiencies, pulmonary infections
Opioid analgesics	Chronic pain	Drowsiness, nausea, constipation, respiratory depression from improper and/or unmonitored prescription, dependence

The comprehensive assessment of pharmacological therapies should result in a consultation report, in which clinically relevant information, properly substantiated by scientific evidence, and concisely descried, should be provided to aid the clinician's decision-making process regarding the prescription of chronic therapies. Actionable suggestions may include drug discontinuation or dose reduction/augmentation, change of frequency of administration, switch to or add-on of a new therapy, as well as monitoring of specific parameters that could serve as indicators of therapeutic benefit or the onset of ADRs. The withdrawing or tapering of a drug should be gradual and in case multiple drugs should be removed this should be done one by one, so to attribute any consequential symptoms to a specific discontinued medication.

### Multidisciplinary pharmacotherapeutic plan

The third phase of medication review and deprescribing involves the collaborative discussion among healthcare professionals such as clinical pharmacologists/hospital pharmacists, who carefully reevaluated polypharmacy and provided a consultation report, and physicians, who finally decide the pharmacological therapy to be prescribed. The patient (or relative/caregiver) should be involved by physicians in the decision-making process concerning prescribed polytherapy. In addition, physicians must document changes in the pharmacological therapy as a result medication review and deprescribing and share accurate and complete information with other healthcare professionals taking care of the patient in different settings (e.g., GPs, nursing home physicians).

### Follow-up

During the fourth and last phase of medication review and deprescribing, the patient's adherence to therapy will be assessed, along with the potential of short-term emergence of symptoms associated with therapy modification, the autonomous cessation/modification of treatments or as prescribed by other doctors, and the onset of ADRs in the short, medium, and long term. For this purpose, possible solutions will include periodic outpatient re-evaluations or home-based telephone monitoring, using a standardized questionnaire to prevent any inter-operator discrepancies. This phase depends on the considered setting and might involve different healthcare professionals.

## Aspects that are specific for different healthcare settings

To ensure the effectiveness of medication review and deprescribing, it is imperative to tailor their implementation to address the specific needs and challenges of various healthcare settings, including primary care, long-term care facilities (LTCFs), hospitals, and palliative care. This section outlines the distinctive aspects of the implementation of medication review and deprescribing in different healthcare settings.

### Primary care

Primary care is one of the most suitable frameworks to conduct medication reviews and starting deprescribing protocols due to the easy and frequent access to GPs practices, particularly for elderly patients. In this setting, the evaluation of pharmacological therapies must be a continuous and dynamic process, where the GP regularly monitors the medications prescribed to the patients and periodically evaluates their effectiveness and safety. A meta-analysis of 33 observational studies evaluating the prevalence of ADRs in primary care reported a mean prevalence ranging from 8.3 to 20.4% [11]. Medication review becomes particularly important when the patient presents new symptoms or diseases, or when there are changes in their clinical situation, and upon returning from emergency room visits or hospital admissions (intervention to improve the accuracy of medication information at transitions). In this setting, telemedicine gives the opportunity to discuss medical conditions and therapies with other specialists and clinical pharmacologists. A systematic review of 58 randomized controlled trials (RCTs) assessing the impact of deprescribing interventions on the reduction of PIPs in primary care showed that antihypertensives, especially diuretics, and nitrates, were the drugs most easily deprescribed, while psychotropic drugs and PPIs were the least deprescribed. The collaboration between GPs and clinical pharmacologists/hospital pharmacists was crucial in providing information to patients/caregivers, thereby ensuring greater effectiveness of deprescribing [12].

In summary, particularly for the elderly, primary care is key for medication review and deprescribing, which are critical when patients' conditions change or after hospital visits. GPs are the healthcare professionals who visit patients more frequently, thus having the opportunity of regularly assessing safety and clinical impacts of these interventions.

### Hospitals

Hospitalisation offers a valuable opportunity to review and address polypharmacy taking into account the patient’s morbidities and goals of care. Approximately 10% of unplanned hospitalizations in geriatric units are caused by ADRs and around 25% of older adults experience at least one ADR in the hospital setting [13–15]. A recent systematic review of the literature reported that preventable ADRs in hospitals account for an average of 37.3% of total ADRs, increasing to over 70% in the elderly population [16, 17]. The resulting economic burden is also considerable, with associated costs during hospital stays ranging from €2,851 to €9,015 per patient [16]. In acute wards, the time needed for physicians to manage the acute medical condition, can sometimes restrict the time available for a thorough medication review. As such, the implementation of medication review and deprescribing with appropriately trained specialists would allow to review patients’ medication regimens upon their admission to the ward (i.e., medication reconciliation), as well as after the patient’s clinical stabilization (e.g., 2–3 days before a scheduled discharge) to possibly deprescribe inappropriate chronic therapies. In this process, ward physicians play a pivotal role, as they oversee the patient’s clinical assessment and decide on the chronic pharmacological therapies at discharge. Beyond pharmacological advice, ward physicians should also collaborate with nurses who can assist in monitoring hospitalized patients and promptly identifying potential drug side effects [18].

A pilot study (FARMACHECK) conducted at the *Fondazione IRCCS Ca’ Granda Ospedale Maggiore Policlinico* in Milan (Italy) demonstrated the effectiveness of an integrated interdisciplinary approach for implementing these services during hospitalization [19]. The study took place in six wards, where trained hospital pharmacists compared home pharmacological therapy with that prescribed in the hospital, within 24–72 h of a patient’s admission, to identify PIPs. The issues found were then discussed with the ward physicians and, before discharge, the pharmacist conducted a final review of the therapies and drafted a discharge letter for the GPs, detailing the reviews and actions taken. The study involved 90 elderly patients, each taking an average of 9.5 medications. During their stay, 455 PIPs were identified, including potential severe DDIs. Approximately two-thirds of the pharmacists' recommendations were accepted, especially those related to contraindicated drugs or potentially major DDIs, and deprescribing was the most frequent action taken following a PIP report [196].

Italian data extensively underscore the need for deprescribing in the hospital setting. The Italian REPOSI registry shows polypharmacy is a relevant problem in internal medicine and geriatrics departments. This registry found that excessive polypharmacy was associated with an increased risk of 1-year mortality [20]. In addition, polypharmacy outperformed sophisticated indicators of quality of therapy, like Beers’ and STOPP criteria and the number of DDIs, as a correlate of primary clinical outcomes. In this setting, previously published data showed no significant effect of PIPs according to Beers' criteria on health outcomes among older adults until 3 months after discharge [17]. For all these reasons, medication review and deprescribing remain a solid tool to reduce excessive drug expenditure and possibly ameliorate clinical outcomes.

Regarding the emergency department, observational studies have shown that ADRs resulting from over-treatment, under-treatment, or PIPs account for 1.5–15% of all emergency admissions, with 50% of them being preventable [21, 22]. In this regard, the Identification of Seniors at Risk (ISAR) is a screening tool that can be employed to identify older adults at risk of adverse functional outcomes, such as hospitalization and death post emergency department admission, thus requiring an urgent need for medication review [23].

The assessment for potential medication review and deprescribing activities can also be performed during an emergency department stay, as over half of the patients are discharged home without subsequent hospital admission. A prospective observational study conducted in a Spanish emergency department described pharmacists-led interventions over a 3-h period from Monday to Friday. They categorized detected therapeutic errors based on relevance and severity using the National Coordinating Council for Medication Error Reporting and Prevention (NCC-MERP) index and the High-Alert Medications Institute for Safe Medication Practices (ISMP) list [24]. During the study, 529 interventions were made for 390 elderly patients, equating to 1.4 interventions per patient, with an 84.9% acceptance rate. Of all potential therapeutic errors, 112 (21.2%) were on the ISMP list of high-alert medications. Using the NCC-MERP severity index, 150 (28.3%) of these errors could lead to adverse clinical outcomes.

In summary, it is important to define the most suitable timeframe for medication review and deprescribing, which might be after the clinical stabilization of the patient. Documenting medication review and deprescribing guarantees seamless therapeutic continuity in primary care. GPs play a crucial role in ensuring therapeutic continuity, managing possible withdrawal symptoms, preventing unintentional re-prescription of previously discontinued therapies, and coordinating the timing of internal or geriatric follow-up visits.

### Long-term care facilities and community hospitals

The implementation of medication review and deprescribing is particularly important in the setting of LCTFs, nursing homes (NHs), and community hospitals, where a significant proportion of patients are on polypharmacy and PIMs are commonly prescribed [25, 26]. Indeed, despite recommendations to limit PIPs, especially regarding antipsychotics, benzodiazepines, antidepressants, PPIs, or antiplatelet agents, these drugs are widely used, often inappropriately, in up to 76% of residents [27, 28]. Furthermore, additional challenges arise in weighing uncertain balance between benefit and risks in patients with limited life expectancy, especially in the case of drugs with potential long-term benefits, such as those used for primary or secondary prevention. It has been observed that lipid-lowering agents and anti-dementia drugs are frequently prescribed even in the terminal stages of a patient's life [29, 30]. In the field of primary care, one of the most prevalent reasons for PIPs is the absence of adequate medication reconciliation by GPs. This issue is often attributed to a conservative approach towards medicines prescriptions made by specialists [31]. Furthermore, a lack of medication review interventions might lead to therapeutic inertia that is a lack of timely adjustment to therapy when a patient's treatment goals are not met, partly due to the limited visit time and the absence of dedicated healthcare professionals for a thorough reassessment of the appropriateness of all medication regimens. In this context, medications which are commonly used among the elderly patients, such as PPIs, non-steroidal anti-inflammatory drugs (NSAIDs), and benzodiazepines are frequently inappropriately prescribed [32]. LCTFs, nursing homes (NHs), and community hospitals are critical healthcare settings for the evaluation of chronic pharmacological therapies under non-acute conditions. In these settings, medicines are administered by healthcare professionals, reducing concerns about adherence and self-medication. Furthermore, physicians have more time to develop therapeutic regimens and to monitor clinical outcomes, such as early symptoms of ADRs, lack of effectiveness, or the emergence of additional diseases [33].

In LTCFs, medication reconciliation should always be conducted upon admission. Subsequently, medical team should undertake medication reviews periodically (e.g., every two months), depending on the facility's capabilities. This consistent schedule ensures appropriate medication management aligned with individual patient needs [33].

A systematic review evaluating the effects of medication review and deprescribing in NHs found these strategies effective in decreasing the number PIPs, leading to reduced fall incidents, and lower mortality risk [34]. Importantly, these interventions resulted safe, with no evidence of symptom rebound or reduced quality of life [35].

A particularly challenging issue in LTCFs is the management of psychotropic drugs, necessitating thorough therapy reviews. A multicenter prospective study in Northern Italy, involving 272 residents across 10 NHs, confirmed the feasibility of deprescribing psychotropic medications. Conducted between September 2013 and May 2014, with 14 voluntary physicians, the study revealed no adverse withdrawal reactions, underscoring the potential of a multidisciplinary and multifaceted strategy in future research for optimizing polypharmacy in this population [27].

In summary, a large body of evidence documented issues concerning PIPs of specific drug classes in LTCFs. This setting allows for close monitoring of treatment adherence, the emergence of any rebound symptoms, and, more in general, the clinical outcomes of medication review and deprescribing.

### Palliative care

According to a Swedish cohort study including 545,212 older people in their last year of life, the proportion of patients with hyper-polypharmacy (i.e., the use of ≥ 10 different medicines) increased from 30 to 50% between the twelfth and the final month of life, as well as the mean number of PIMs, that increased from 8 to 10% [35]. Another observational study, involving a cohort of 372 patients with advanced-stage cancer receiving palliative care, reported that 22% of patients were prescribed at least one PIM [36].

Critical factors in medication review and deprescribing among patients receiving palliative care include reduced life expectancy, a shift in therapeutic goals from long-term prevention to symptom control, the time required for drugs to exert benefits, medicines administration difficulties, risks associated with the sudden discontinuation of certain medications (such as corticosteroids and long-acting benzodiazepines), and psychosocial factors. It is advisable to discontinue one medication at a time, particularly with cardiovascular drugs, and only after gradual dose adjustments, to minimize the risk of withdrawal effects, which are a significant concern for patients and their families.

Specific drug classes require a particular evaluation for deprescribing, including antibiotics, antihypertensives, and antidiabetics. The use of antibiotics should be assessed for expected effectiveness, symptoms relief (e.g., fever reduction), potential ADRs, and costs (including the impact on microbial resistance). Strictly controlling blood pressure or blood glucose levels does not provide substantial benefits at the end of life, but it could, instead, deteriorate the patient’s quality of life, lower the pharmacological adherence, increase the likelihood of administration errors, and heighten the risk of ADRs.

As for LTCFs, medication reconciliation should always be conducted upon admission, and regular medication reviews should be performed periodically.

The OncPal Deprescribing Guidelines is an assessment tool to evaluate prescribing appropriateness, developed through a consensus of key experts in the field. This tool consists of a list of PIMs specific to palliative oncology, along with the rationale for each selection [37]. Adopting a multidimensional approach, it is crucial to involve a palliative care physician in medication review and deprescribing, aligning with the patient's treatment goals. The multiprofessional team should also include nurses, who can help in monitoring the patients’ clinical conditions and identifying any withdrawal symptoms, and psychologists, who assist in communicating therapeutic objectives with patients. Importantly, this process should include the patient and caregivers, ensuring shared decision-making, respecting patient autonomy, and providing transparency and support for any doubts or uncertainties.

In an Italian study conducted on 589 patients in hospice care, researchers calculated the prevalence of patients receiving preventive medications (deemed inappropriate) and those receiving drugs for symptoms management drugs (considered appropriate) at both admission and death [38]. A reduction in the mean number of medications—from 9.7 at admission to 8.7 at death—was observed. A significant decrease in the proportion of patients receiving at least one preventive medication was observed, from 87% upon admission to 49% at the time of death. Generally, it has been observed that the practices between different hospices can vary significantly, likely depending on the palliative care physician in charge of the patients [38].

In summary, in palliative care, it is important to re-evaluate the risk–benefit profile of chronic medications considering the reduced life expectancy, and to actively involve the patient/caregivers in the decision-making process.

## Challenges and potential solutions for the implementation of medication review and deprescribing

Although the implementation of medication review and deprescribing is essential particularly for aging populations and those with complex medical needs, this process must face numerous challenges in clinical practice. One of the main obstacles is the reluctance of patients and their families in changing or discontinuing medications that have been used for a long time or were prescribed by a specialist, due to the fear of symptoms rebound. In addition, in all healthcare settings, physicians often have limited time to conduct comprehensive medication reviews. Patients with multimorbidity usually have complex medication regimens, making medication review and management difficult. More importantly, effective deprescribing requires collaboration among different healthcare professionals, including physicians (e.g., geriatricians, internists, GPs), clinical pharmacologists or pharmacists, and nurses. A large body of evidence documented that the involvement of the clinical pharmacologist in the multidisciplinary team is associated with significantly improved efficacy of the intervention and improved clinical outcomes [39]. The role of hospital and community pharmacists has been proved to be considerably valuable for the implementation of medication review interventions, especially for the follow-up and in the transition of care [40, 41].

Furthermore, patients may not have complete information about their medications, thus potentially limiting the accuracy of medication reviews. Following deprescribing, monitoring patients for any ADRs or adverse health outcomes is essential [42].

Several potential solutions can address the above-mentioned challenges. Explaining the potential benefits of deprescribing, such as reducing medication-related side effects and improving quality of life, as well as promoting shared decision-making, is essential to actively involve patients in the process. On the other hand, establishing clear protocols, guidelines, and workflows for medication review and deprescribing to be shared with all healthcare professionals is necessary for best clinical practice. Maintaining an up-to-date list of their medications, including over the counter and herbal supplements, as well as investing in patient education about their medications and potential side effects represent other important solutions.

In conclusion, overcoming these challenges demands a patient-centred and collaborative approach among physicians, clinical pharmacologists or pharmacists, nurses, and patients/caregivers [43].

## Evaluation of the impact of medication review and deprescribing

Effective implementation of medication review and deprescribing in different healthcare settings may have a significant impact, both clinically and economically. Accordingly, a reduction in the number of medications taken by patients is expected, resulting in better tolerability, increased therapeutic adherence and improved clinical outcomes [6, 8].

The effects of medication review and deprescribing can be evaluated as pre-/post-intervention analyses, comparing the periods before and after the intervention, through the analysis of real-world data. Types of outcomes to be investigated include clinical outcomes (e.g., patient hospitalization rate, rate of ADR), outcomes of process (e.g., appropriate prescribing and frequency of PIPs, discontinuation of prescribed medications and/or resumption of previous therapy by outpatient visits or home monitoring, level of adherence to chronic therapies), patient-reported outcomes (e.g., patient-reported experience and patient-reported outcome measures), and economic outcomes (e.g., health care expenditures related to hospitalization due to ADRs or prescription drugs). Clinical and economic endpoints should be evaluated at defined time points, with a short-, medium- and long-term perspective [44].

## Conclusions

Medication review and deprescribing in different clinical settings represents a crucial activity to improve both the quality of care and quality of life of patients on polypharmacy, reducing medication burden and related risk of DDIs and ADRs. Key elements to be considered for the successful implementation of medication review and deprescribing consist of the proper choice of tools, timing, and healthcare professionals to be involved. To analyze the clinical and economic impact of medication review and deprescribing in different healthcare settings is urgently needed to appropriately allocate resources and properly such a service training physicians and other healthcare professionals.
